# Supplementary material for: Dimensions of Cellulose Nanocrystals from Cotton and Bacterial Cellulose: Comparison of Microscopy and Scattering Techniques
Source: Nanomaterials (Basel). 2024 Feb 29;14(5):455. doi: 10.3390/nano14050455 (PMC10934643; doi:10.3390/nano14050455)
Supplement: Supplementary file 1 [file nanomaterials-14-00455-s001.zip › nanomaterials-2876660-supplementary.pdf]

Supporting information

Dimensions of Cellulose Nanocrystals from Cotton and Bacterial Cellulose: Comparison of Microscopy and Scattering Techniques

Vladimir Grachev [1], Olivier Deschaume [2], Peter R. Lang [3], Minne Paul Lettinga [2,3], Carmen Bartic [2] and Wim Thielemans [1,*]

[1] Sustainable Materials Lab, Department of Chemical Engineering, KU Leuven, Campus Kulak Kortrijk, Etienne Sabbelaan 53, 8500 Kortrijk, Belgium; vladimir.grachev@kuleuven.be

[2] Laboratory for Soft Matter Physics and Biophysics, Department of Physics and Astronomy, KU Leuven, Celestijnenlaan 200D Box 2416, 3001 Leuven, Belgium; olivier.deschaume@kuleuven.be (O.D.); pavlik.lettinga@kuleuven.be (M.P.L.); carmen.bartic@kuleuven.be (C.B.)

[3] Institute for Biomacromolecular Systems and Processes Group (IBI-4), Forschungszentrum Jülich, Wilhelm-Johnen-Straße, 52428 Jülich, Germany; p.lang@fz-juelich.de

* Correspondence: wim.thielemans@kuleuven.be

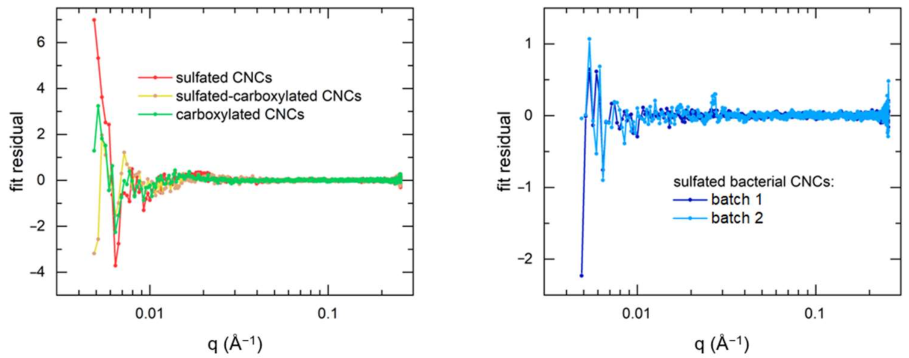

Figure S1. SAXS fit residual plots of cotton CNCs (left) and bacterial CNCs (right)

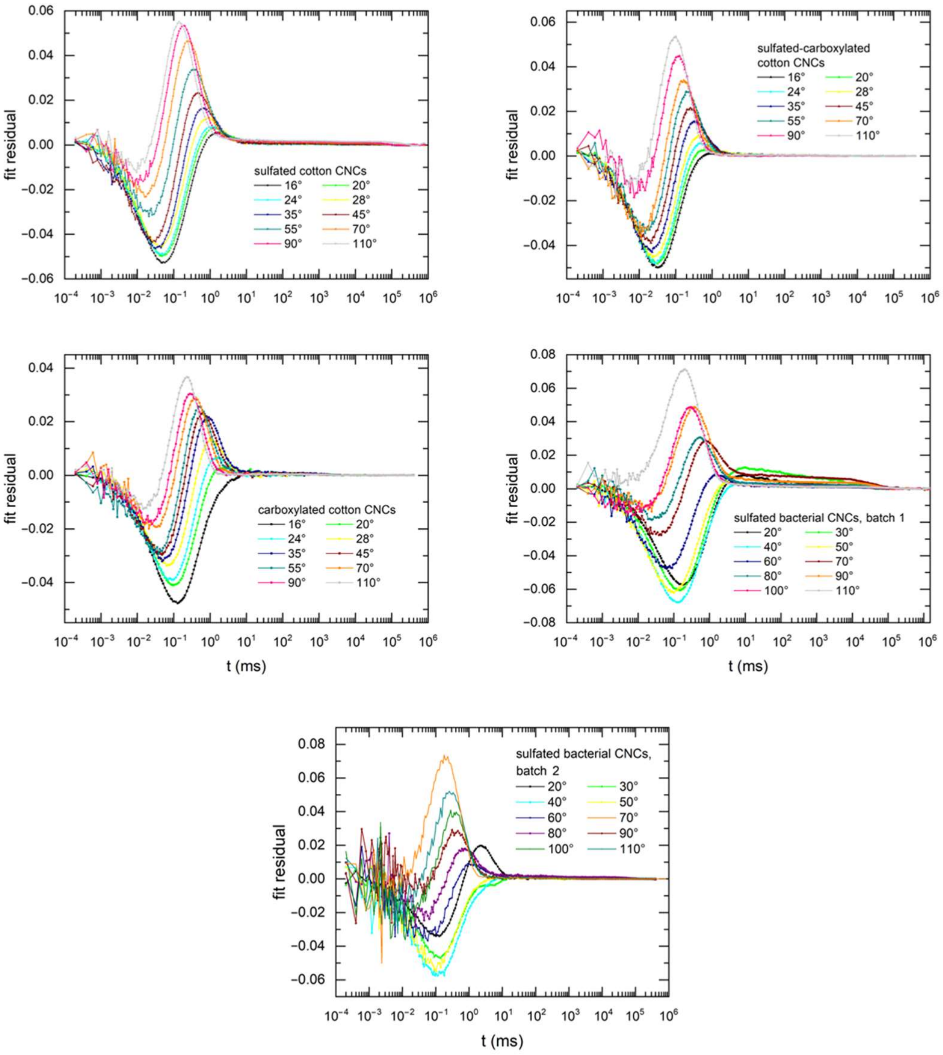

Figure S2. DDLS fit residuals of cotton and bacterial CNCs

Table S1. Experimental and calculated translational and rotational diffusion coefficients of sulfated cotton CNCs

	$\langle D_{tr} \rangle$ * 10^8, cm²/s			$\langle D_{rot} \rangle$, Hz		
d_{hd}, nm	42 ($\langle b \rangle$)	24.4 ($\frac{\langle b \rangle + \langle h \rangle}{2}$)	6.8 ($\langle h \rangle$)	42 ($\langle b \rangle$)	24.4 ($\frac{\langle b \rangle + \langle h \rangle}{2}$)	6.8 ($\langle h \rangle$)
experimental		**6.7±0.1**			**286±7**	
$\sigma_L = 0.1$	4.5±0.1	5.7±0.1	8.6±0.1	572±1	821±1	1466±1
$\sigma_L = 0.3$	5.2±0.1	6.7±0.1	10.5±0.1	416±2	582±3	1005±6
$\sigma_L = 0.5$	5.6±0.1	**7.1±0.1**	11.2±0.2	231±5	**309±7**	501±13
$\sigma_L = 0.8$	4.7±0.1	5.8±0.1	8.5±0.2	75±5	93±6	138±10

Table S2. Experimental and calculated translational and rotational diffusion coefficients of sulfated-carboxylated cotton CNCs

	$\langle D_{tr} \rangle$ * 10^8, cm²/s			$\langle D_{rot} \rangle$, Hz		
d_{hd}, nm	38 ($\langle b \rangle$)	23.9 ($\frac{\langle b \rangle + \langle h \rangle}{2}$)	7.8 ($\langle h \rangle$)	38 ($\langle b \rangle$)	23.9 ($\frac{\langle b \rangle + \langle h \rangle}{2}$)	7.8 ($\langle h \rangle$)
experimental		**9.3±0.2**			**462±10**	
$\sigma_L = 0.1$	4.5±0.1	5.6±0. 1	7.9±0.1	523±1	715±1	1156±1
$\sigma_L = 0.3$	5.1±0.1	6.4±0.1	9.4±0.1	379±2	507±4	796±6
$\sigma_L = 0.5$	5.4±0.1	6.8±0.1	**9.8±0.2**	211±5	272±7	**407±12**
$\sigma_L = 0.8$	4.5±0.1	5.5±0.1	7.6±0.2	69±5	84±6	117±8

Table S3. Experimental and calculated translational and rotational diffusion coefficients of carboxylated cotton CNCs

	$\langle D_{tr} \rangle$ * 10^8, cm²/s			$\langle D_{rot} \rangle$, Hz		
d_{hd}, nm	40 ($\langle b \rangle$)	24 ($\frac{\langle b \rangle + \langle h \rangle}{2}$)	8 ($\langle h \rangle$)	40 ($\langle b \rangle$)	24 ($\frac{\langle b \rangle + \langle h \rangle}{2}$)	8 ($\langle h \rangle$)
experimental		**4.7±0.1**			**123±5**	
$\sigma_L = 0.1$	3.6±0.1	4.4±0.1	6.1±0.1	245±1	324±1	505±1
$\sigma_L = 0.3$	3.9±0.1	4.8±0.1	6.7±0.1	182±2	237±3	360±5
$\sigma_L = 0.5$	3.9±0.1	**4.8±0.1**	6.7±0.1	108±4	**136±5**	199±8
$\sigma_L = 0.8$	3.3±0.1	3.9±0.1	5.3±0.1	40±3	48±3	66±5

Table S4. Experimental and calculated translational and rotational diffusion coefficients of sulfated bacterial CNCs (batch 1)

	$\langle D_{tr} \rangle * 10^8$, cm²/s			$\langle D_{rot} \rangle$, Hz		
d_{hd}, nm	40 ($\langle b \rangle$)	23.5 ($\frac{\langle b \rangle + \langle h \rangle}{2}$)	6.9 ($\langle h \rangle$)	40 ($\langle b \rangle$)	23.5 ($\frac{\langle b \rangle + \langle h \rangle}{2}$)	6.9 ($\langle h \rangle$)
experimental	**3.9±0.1**			**43±2**		
σ_L = 0.1	2.9±0.1	3.5±0.1	4.9±0.1	114±1	146±1	226±1
σ_L = 0.3	2.9±0.1	3.5±0.1	4.9±0.1	91±2	116±2	176±3
σ_L = 0.5	2.8±0.1	3.4±0.1	4.7±0.1	61±2	76±3	112±4
σ_L = 0.8	2.4±0.1	2.8±0.1	**3.8±0.1**	26±1	31±2	**43±2**

Table S5. Experimental and calculated translational and rotational diffusion coefficients of sulfated bacterial CNCs (batch 2)

	$\langle D_{tr} \rangle * 10^8$, cm²/s			$\langle D_{rot} \rangle$, Hz		
d_{hd}, nm	39 ($\langle b \rangle$)	23.4 ($\frac{\langle b \rangle + \langle h \rangle}{2}$)	7.8 ($\langle h \rangle$)	39 ($\langle b \rangle$)	23.4 ($\frac{\langle b \rangle + \langle h \rangle}{2}$)	7.8 ($\langle h \rangle$)
experimental	**3.7±0.04**			**44±3**		
σ_L = 0.1	3.0±0. 1	3.6±0.1	4.9±0.1	124±1	159±1	237±1
σ_L = 0.3	3.1±0.1	3.6±0.1	4.9±0.1	99±2	125±3	182±4
σ_L = 0.5	3.0±0.1	3.5±0.1	4.7±0.1	65±3	80±3	114±5
σ_L = 0.8	2.5±0.1	2.9±0.1	**3.9±0.1**	27±2	32±2	**43±3**
